# Oral verruciform xanthomas mimicking potentially malignant disorders

**DOI:** 10.4317/jced.61411

**Published:** 2024-04-01

**Authors:** Kaique-Alberto Preto, Gabriela Lopes-Santos, Marcelo-Junior Zanda, Denise-Tostes Oliveira

**Affiliations:** 1 DDS. Department of Surgery, Stomatology, Pathology and Radiology, Bauru School of Dentistry, University of São Paulo, Bauru, São Paulo, Brazil; 2 DDS, MSc. Department of Surgery, Stomatology, Pathology and Radiology, Bauru School of Dentistry, University of São Paulo, Bauru, São Paulo, Brazil; 3 DDS, MSc, PhD. Clinical Research Center, Bauru School of Dentistry, University of São Paulo, Bauru, São Paulo, Brazil; 4 DDS, MSc, PhD. Department of Surgery, Stomatology, Pathology and Radiology, Bauru School of Dentistry, University of São Paulo, Bauru, São Paulo, Brazil

## Abstract

Verruciform xanthoma represents a reactive lesion, common in the skin and somewhat rare in the mouth. Cases description: Two cases of verrucous white plaques, located on the tongue and the floor of mouth of different 30-years-old man and woman and clinically diagnosed as leukoplakia, are described. The histopathological analyses confirmed the diagnosis of oral verruciform xanthomas for both lesions. Practical implications: Despite of uncommon in the oral cavity, the verruciform xanthoma, particularly when affecting regions with a higher risk of developing oral cancer, should be included in differential diagnosis of oral potentially malignant disorders. The histopathological analysis remains as “gold standard technique” for a more accurate diagnosis of oral verruciform xanthoma.

** Key words:**Foam cells, Verruciform xanthoma, Leukoplakia, Tongue.

## Introduction

Oral verruciform xanthoma is an uncommon reactive lesion, characterized by papules and/or plaques with variable surfaces (papillary, flat, verrucous) and colors (red, pink, yellowish, or brownish) and occasionally, white verrucous/hyperkeratotic appearance (1,2). Although somewhat rare in the mouth, it occurs as an asymptomatic solitary lesion (2mm to 1.5 cm in size), sessile or pedunculated presenting a slow growth. The majority of oral verruciform xanthomas are detected as an incidental finding in masticatory mucosa of the palate and gingiva, but they also found in other unusual locations such as tongue, floor of mouth and lip (3-7).

Oral verruciform xanthoma can easily be misinterpreted as a precursor of the highest prevalent potentially malignant lesion (8) e.g., leukoplakia. It is well-established that leukoplakia has a questionable malignant risk, and its accurate diagnosis requires ruling out other oral white lesions with specific etiology (8). Consequently, the histopathological analysis is crucial in white lesions for identification of dysplastic changes in the squamous epithelium that increase the risk of oral cancer development.

The aim of this article is reported two cases of oral verrucous xanthomas that had clinical features of leukoplakia, an oral potentially malignant lesion.

## Case Report

-Case 1 

**Figure 1:**
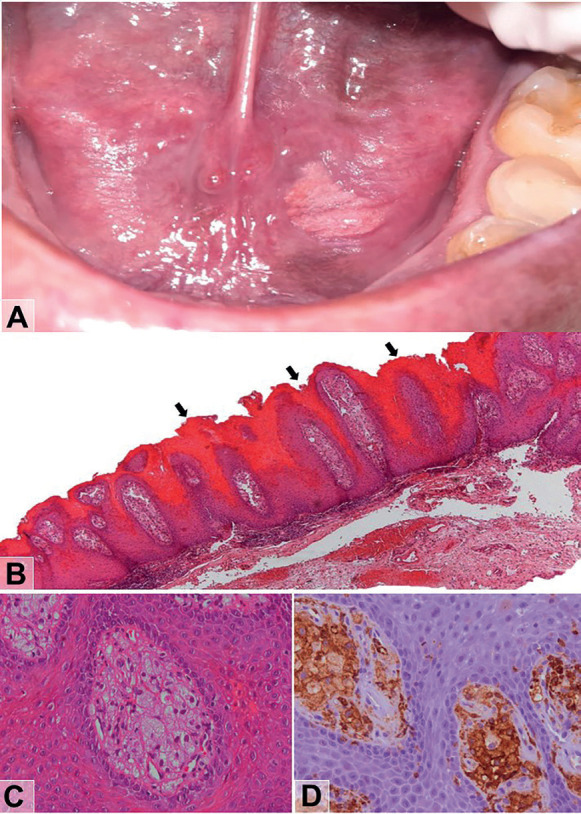
A) White plaque with verrucous surface and uniform appearance, well-defined borders measuring approximately 1 cm in diameter localized on the floor of the mouth. B) The prominent papillary lesion showing abundant keratin on surface of the hyperplastic oral squamous stratified epithelium (described as “wart on fire” – black arrow) forming elongated rete pegs. In the connective tissue papillae, the presence of macrophages with foamy cytoplasm (xanthoma cells) and some eccentrically nuclei were observed (C). Additionally, an intense chronic inflammatory infiltrate was also found in connective tissue. The immunohistochemical staining confirmed that foamy macrophages were positive for CD163 (D). The final diagnosis was verruciform xanthoma. Original magnification, Hematoxylin and Eosin: B = ×50; C = ×200; Immunohistochemistry: D = CD163 —×200).

A 30-year-old male, smoker, was referred by his dentist due to a verrucous white plaque in the oral mucosa. During the physical examination, an unremovable white plaque slightly elevated, with well-defined borders, verrucous surface, and uniform appearance, asymptomatic, measuring approximately 1 cm in diameter, was identified on the floor of the mouth (Fig. 1A). No local or systemic causes were associated with the lesion. His medical history was unremarkable. The clinical diagnosis was leukoplakia. The lesion was surgically excised, and the material sent for histopathological analysis. Microscopic sections, stained in hematoxylin and eosin, revealed prominent papillary lesion showing abundant keratin on surface of the hyperplastic oral squamous stratified epithelium forming elongated rete pegs, typically described as “wart on fire” (9) (Fig. 1B). There is no evidence of epithelial dysplasia. Subjacent, in the connective tissue papillae, the presence of macrophages with foamy cytoplasm (xanthoma cells) and some eccentric nuclei were observed (Fig. 1C). The immunohistochemical staining confirmed that foamy macrophages were positive for CD163 (Fig. 1D) and negative for S100. Additionally, an intense chronic inflammatory infiltrate was also found in connective tissue. The final diagnosis was verruciform xanthoma. The patient was oriented about his lesion and no signs of recurrence were detected after six months of follow-up.

-Case 2 

**Figure 2:**
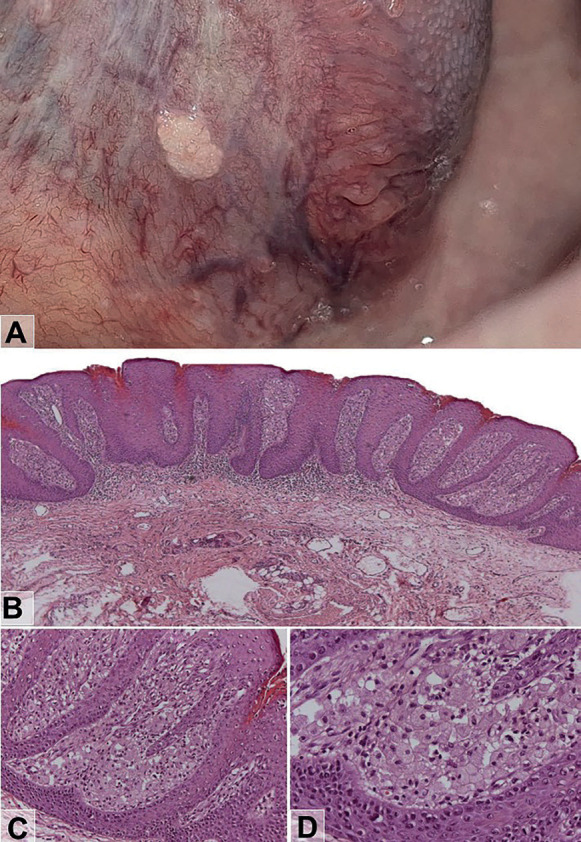
A) Homogenous white plaque with verrucous surface, well-defined contour, measuring approximately 1.5 cm in diameter located in the posterior region of the ventral tongue. B) The papillary architecture presenting hyperplastic and hyperkeratotic squamous stratified epithelium with elongated epithelial ridges. C and D) Numerous foamy macrophages with a granular cytoplasmic appearance were observed within the connective tissue papillae. An intense subepithelial mononuclear inflammatory infiltrate was also found. The diagnosis was verruciform xanthoma. Original magnification, Hematoxylin and Eosin: B = ×50; C = ×200; D = ×400).

A 30-year-old woman, non-smoker, was referred to the dentist for evaluation of a lesion in the tongue. Intraoral examination revealed a homogenous white plaque with verrucous surface, well-defined contour, measuring approximately 1.5 cm in diameter, asymptomatic, located in the posterior region of the ventral tongue (Fig. 2A). The patient reported that the lesion had been present for approximately one year. The clinical diagnosis was leukoplakia. The lesion was treated by excisional biopsy. The histopathological analysis revealed a lesion with papillary architecture presenting hyperplastic and hyperkeratotic squamous stratified epithelium with elongated epithelial ridges (Fig. 2B) and without signs of epithelial dysplasia. Numerous foamy macrophages with a granular cytoplasmic appearance were observed within the connective tissue papillae (Fig. 2C,D). An intense subepithelial mononuclear inflammatory infiltrate was also found. The diagnosis established was verruciform xanthoma. The patient was monitored for 5 months, and no clinical signs of lesion recurrence were detected.

## Discussion

Although there are many cases of oral verruciform xanthoma reported since the 1970s in English literature, this lesion remains little known by health professionals worldwide (3,4).

Clinically, as reported in our patients, the presence of white verrucous plaque in oral mucosa, asymptomatic and not associated with specific etiologic factors may be indistinguishable from leukoplasia, an oral potentially malignant disorder. Furthermore, the differential diagnosis of this lesion includes other oral verrucous lesions such as squamous papilloma, verrucous carcinoma or squamous cell carcinoma (10).

Particularly if the verruciform xanthoma manifests in smoker patients and/or in oral regions predisposed to elevated risk for cancer development as in our reported cases, its clinical diagnosis may be suggestive of malignancy. In addition, despite its benign appearance, good prognosis and unclear pathogenesis, oral verruciform xanthoma may occur simultaneously with other lesions or malignancies such as oral squamous cell carcinoma or carcinoma *in situ* (10).

Then, the histopathological analysis is required for diagnosis of oral verrucous xanthoma, especially in smoker patients presenting white verrucous lesions on the tongue or floor of mouth. Microscopically, the presence of verrucous proliferation of squamous epithelium with elongated rete ridges and markedly thickened parakeratin associated with macrophages with foamy cytoplasm due to the presence of lipids (xanthoma cells) are classical morphologic characteristics found in xanthoma (9), such as observed in present cases reported. The immunohistochemical stains, including positivity for CD68, CD63, or CD163, as used in one of our patients, are not essential for the diagnosis of oral xanthoma but contribute to confirm the macrophage origin of the foamy cells (4).

In conclusion, verruciform xanthoma is an uncommon oral lesion with clinical similarities to oral potentially malignant disorder and should be included in differential diagnosis of verrucous lesions, particularly when it affects regions with a higher risk of developing oral cancer in smoker patients. Then, the histopathological analysis remains as “gold standard technique” for more accurate diagnosis of oral verruciform xanthoma, due to its classical microscopic features.
